# Efficient and transgene-free genome editing in wheat through transient expression of CRISPR/Cas9 DNA or RNA

**DOI:** 10.1038/ncomms12617

**Published:** 2016-08-25

**Authors:** Yi Zhang, Zhen Liang, Yuan Zong, Yanpeng Wang, Jinxing Liu, Kunling Chen, Jin-Long Qiu, Caixia Gao

**Affiliations:** 1 State Key Laboratory of Plant Cell and Chromosome Engineering, Institute of Genetics and Developmental Biology, Chinese Academy of Sciences, Beijing 100101, China; 2 University of Chinese Academy of Sciences, Beijing 100049, China; 3 State Key Laboratory of Plant Genomics, Institute of Microbiology, Chinese Academy of Sciences, Beijing 100101, China

## Abstract

Editing plant genomes is technically challenging in hard-to-transform plants and usually involves transgenic intermediates, which causes regulatory concerns. Here we report two simple and efficient genome-editing methods in which plants are regenerated from callus cells transiently expressing CRISPR/Cas9 introduced as DNA or RNA. This transient expression-based genome-editing system is highly efficient and specific for producing transgene-free and homozygous wheat mutants in the T0 generation. We demonstrate our protocol to edit genes in hexaploid bread wheat and tetraploid durum wheat, and show that we are able to generate mutants with no detectable transgenes. Our methods may be applicable to other plant species, thus offering the potential to accelerate basic and applied plant genome-engineering research.

## Abstract (web summary)

Plant genome editing typically relies upon transgenic intermediates, which is a concern given the current regulatory requirements concerning GMOs. Here, Zhang *et al*. describe a method to edit wheat genomes by transiently expressing CRISPR/Cas9 DNA or RNA, and are able to generate mutant plants with no detectable transgenes.

## Graphical abstract

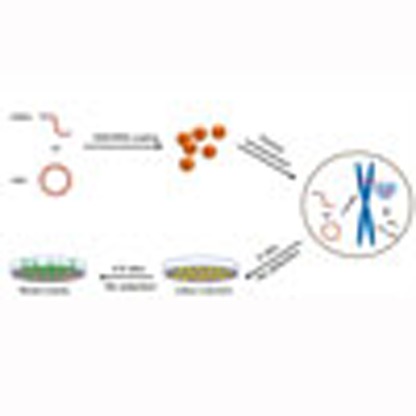

The CRISPR (clustered regularly interspaced short palindromic repeats)/associated nuclease Cas9 system has been widely used in plants to introduce targeted mutations for studying gene function and providing new avenues for crop improvement^1^,^2^. Typically, CRISPR/Cas9 expression cassettes are delivered to plant cells and expressed, which cleaves chromosomal target sites and produces site-specific DNA double-strand breaks, leading to genome modifications during the repair process^3^. However, it is still challenging to create mutations in some transformation-recalcitrant species, such as wheat, soybean, sorghum, cotton and woody plants. Moreover, the introduced CRISPR/Cas9 DNA usually becomes integrated into plant chromosomes, increasing the chance of off-target changes^4^ and causing legislation concerns about genetically modified organisms^5^. Consequently, substantial efforts are being devoted to overcome the above problems. Recently, Woo *et al*.^6^ achieved plant genome editing by delivering pre-assembled CRISPR/Cas9 ribonucleoproteins to protoplasts of lettuce, and successfully obtained transgene-free mutant plants. However, for most plant species, the isolation and culture of protoplasts is cumbersome, and in most monocotyledons, especially major cereal crops, regeneration of plants from cultured protoplasts is still not feasible.

Here we demonstrate a simple and efficient genome-editing approach by which mutant plants are regenerated after transiently expressing CRISPR/Cas9 DNA (abbreviated as TECCDNA), or through transient expression of the *in vitro* transcripts (IVTs) of Cas9-coding sequence and guide RNA (TECCRNA hereafter), into wheat callus cells. Furthermore, our tissue culture procedures are free of lengthy, costly and labour-intensive herbicide selection (Fig. 1), and homozygous mutant plants with no detectable transgenes are identified in T0 populations. We found that these genome-editing methods are highly efficient for both hexaploid bread wheat (*Triticum aestivum* L., AABBDD, 2*n*=6*x*=42) and tetraploid durum wheat (*T. turgidum* L. var. *durum*, AABB, 2*n*=4*x*=28), and should be applicable to other plant species.

## Results

### Development of the TECCDNA method

**Figure 1:**
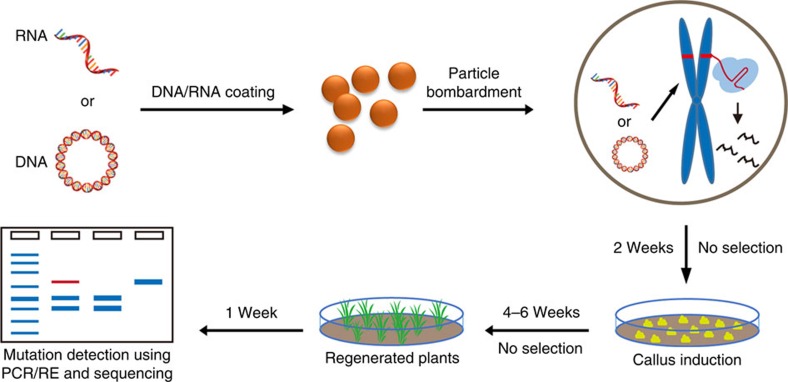
Overview of the genome-editing methods based on TECCDNA or TECCRNA. The CRISPR/Cas9 DNA (plasmid constructs) or RNA (*in vitro* synthesized transcripts) is delivered into immature wheat embryos by particle bombardment. After transient expression and function, CRISPR/Cas9 DNA or RNA becomes degraded, while the bombarded embryos produce callus cells from which seedlings are regenerated. The T0 seedlings are examined using PCR-RE assay and DNA sequencing to identify targeted mutants.

**Figure 2:**
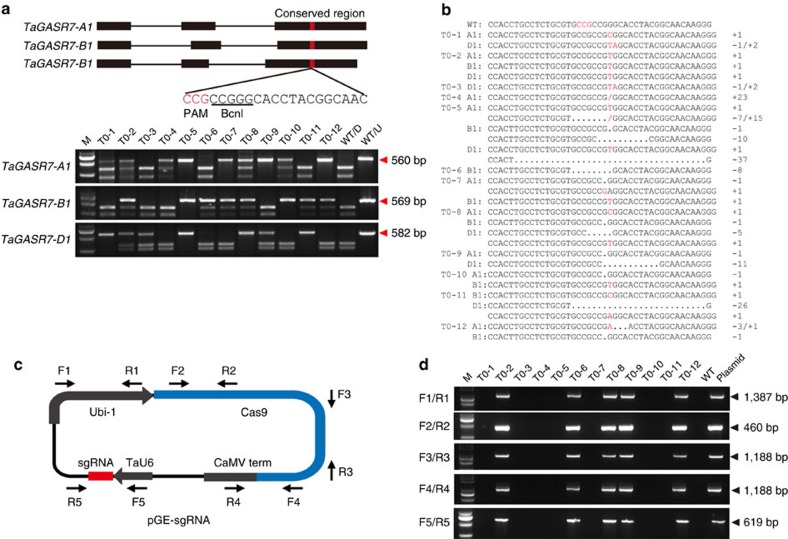
Development and validation of the TECCDNA method. (**a**) Sequence of an sgRNA designed to target a site within a conserved region of exon 3 of *TaGASR7* homoeologues. The protospacer-adjacent motif (PAM) sequence is highlighted in red and the *BcnI* restriction site is underlined. The outcome of PCR-RE assays analysing 12 representative *tagasr7* mutants is shown. Lanes T0-1 to T0-12 show blots of PCR fragments amplified from independent wheat plants digested with BcnI. Lanes labelled WT/D and WT/U are PCR fragments amplified from WT plants with and without BcnI digestion, respectively. The bands marked by red arrowheads are caused by CRISPR/Cas9-induced mutations. (**b**) Genotypes of 12 representative mutant plants identified by sequencing. (**c**) Schematic of the structure of the pGE-sgRNA vector and five primer sets used for detecting transgene-free mutants. sgRNA refers to sgRNAs targeting *TaGASR7, TaDEP1, TaNAC2, TaPIN1, TaLOX2, TdGASR7* and *TaGW2*, respectively. (**d**) Outcome of tests for transgene-free mutants using five primer sets in 12 representative *tagasr7* mutant plants. Lanes without bands identify transgene-free mutants. Lanes labelled WT and plasmid show the PCR fragments amplified from a WT plant and the pGE-TaGASR7 vector, respectively.

First, we developed the TECCDNA-based genome-editing method by working with the three homoeologues of *TaGASR7* (*TaGASR7-A1, -B1* and *-D1*), which have been implicated in the control of grain length and weight^7^. Each of the three homoeologues has three exons and two introns (Fig. 2a), and we designed 5 single guide RNAs (sgRNAs) that target exon 3 because it is highly conserved. After initial testing of nuclease activity in protoplasts^8^, the most effective sgRNA expression cassette (Supplementary Fig. 1 and Supplementary Table 1) was combined with that of Cas9 in a single DNA construct (pGE-TaGASR7, Fig. 2c). This was introduced by particle bombardment into immature embryos of two bread wheat varieties (Bobwhite and Kenong199). Embryogenic calli developed in 2 weeks, and a large number of seedlings (2–3 cm high) were regenerated in another 4–6 weeks. In contrast with most plant genome-editing experiments, we did not add herbicide or antibiotics to the medium to select for transformed plants (Fig. 1). Under our selection-free conditions, the total time from particle bombardment to obtaining testable plants was 6–8 weeks, which is 2–4 weeks shorter than the genome-editing protocol reported previously^9^.

We analysed the sgRNA target site in the regenerated T0 seedlings using PCR restriction enzyme digestion assay (PCR-RE assay)^8^, first using a conserved primer set (Supplementary Table 2) that recognizes all three *TaGASR7* homoeologues and then with three primer pairs specific for each homoeologue (Supplementary Table 2). The mutagenesis frequency was defined as the number of plants carrying the observed mutations over the total number of bombarded immature embryos. For example, from 1,600 bombarded immature embryos of Bobwhite, a total of 80 *tagasr7* mutants (an efficiency of 5.0%) with indels in the targeted region and another set of 21 such mutants from 800 bombarded immature embryos (2.6%) of Kenong199 were identified (Table 1). Targeted mutations were observed in all three homoeologues (Fig. 2a,b). Among the 80 Bobwhite mutants we identified nearly all combinations of mutations involving three *TaGASR7* homoeologues, including 51 mutants with targeted indels in all three genomes (Supplementary Table 3). Importantly, 8 of these 51 mutants had all six alleles simultaneously knocked out (Supplementary Table 3). These data suggest that our new experimental scheme, based on TECCDNA in callus cells, is highly efficient in generating targeted mutations of *TaGASR7* in T0 populations.

### Validation of the TECCDNA method

Next, we examined whether the TECCDNA method was generally applicable for other wheat genes. We targeted the wheat orthologues of rice *DEP1*, *NAC2* and *PIN1* and a wheat lipoxygenase gene (*TaLOX2*). In rice, *DEP1* controls inflorescence architecture and affects panicle growth and grain yield^10^; *NAC2* has been found to regulate shoot branching^11^, and *PIN1* is required for auxin-dependent adventitious root emergence and tillering^12^. *TaLOX2* is highly expressed during grain development and may affect the storability of wheat grains^13^. We developed CRISPR/Cas9 DNA constructs for each of the four genes (Fig. 2c, Supplementary Fig. 1 and Supplementary Table 1), and bombarded immature embryos of Kenong199. The regenerated seedlings were analysed for mutations with conserved and specific primer sets (Supplementary Table 2) for *TaDEP1.* For simplicity, we designed only conserved primers (Supplementary Table 2) to detect mutations in *TaNAC2*, *TaPIN1* and *TaLOX2,* the latter of which exists as a single copy (*TaLOX2-D1* in the D genome). Targeted mutations were easily identified in all four genes in the T0 seedlings using PCR-RE analysis (Supplementary Figs 2 and 3). The mutagenesis frequency varied from 1.0 to 9.5%, and we identified 34 *talox2*-*dd* homozygous mutants among 76 mutant plants (44.7%; Table 1).

### Applicability of TECCDNA method in durum wheat

In addition to bread wheat, durum wheat is also an important crop widely used for pasta foods. Because *GASR7* is highly conserved in tetraploid and hexaploid wheat, the new method was also introduced into two different durum wheat varieties for inducing mutations in *TdGASR7-A1* and *-B1*. The mutagenesis frequencies in tetraploid wheat lines exceeded 1.0%, and homozygous mutants resulting from simultaneous editing of all four alleles were obtained in T0 seedlings (Table 1 and Supplementary Fig. 4). To our knowledge, successful genome editing has not been reported for tetraploid wheat before this work. Clearly, TECCDNA-based genome editing provides a reliable genome-engineering method for this important crop.

### Detection of transgenes

Because the mutant seedlings were developed in the absence of herbicide selection, there is a high probability that the CRISPR/Cas9 DNA construct is not integrated in the genome. This was investigated by examining the presence of plasmid DNA in the T0 mutants using PCR. We designed primer sets (Supplementary Table 2) specific for five discrete regions in the CRISPR/Cas9 DNA construct, representing all major parts (Fig. 2c). On the basis of the PCR analysis, the CRISPR/Cas9 DNA construct was found to be absent in 43.8% (cv Bobwhite; Fig. 2d) and 61.9% (cv Kenong199) of the T0 mutants for *TaGASR7* (Table 1). In addition, we conducted Southern blot analysis for three independent *tagasr7* mutants, without (T0-3 and T0-10) or with (T0-12) CRISPR/Cas9 DNA as detected with the above PCR assay. As anticipated, no hybridization signals were found in T0-3 and T0-10, although hybridizing bands were detected in T0-12 (Supplementary Fig. 5). Therefore, we could obtain some homozygous *tagasr7* mutants with no detectable transgenes in the T0 generation (Table 1 and Supplementary Table 3). For the other four genes, our PCR analysis showed that the frequencies of mutants without detectable transgenes were 53.8% (*TaDEP1*), 75.0% (*TaNAC2*), 62.5% (*TaPIN1*) and 86.8% (*TaLOX*2) (Table 1). Likewise, absence of CRISPR/Cas9 DNA integration was found in 54.5–58.3% of the T0 mutants of the two durum wheat varieties (Table 1). Homozygous mutants with no detectable transgenes were also identified in *TaLOX*2 and *TdGASR7* (Table 1). Thus, plants with targeted mutations and lacking active transgenes can be efficiently obtained using our TECCDNA genome-editing method.

### Developing and testing the TECCRNA method

**Figure 3:**
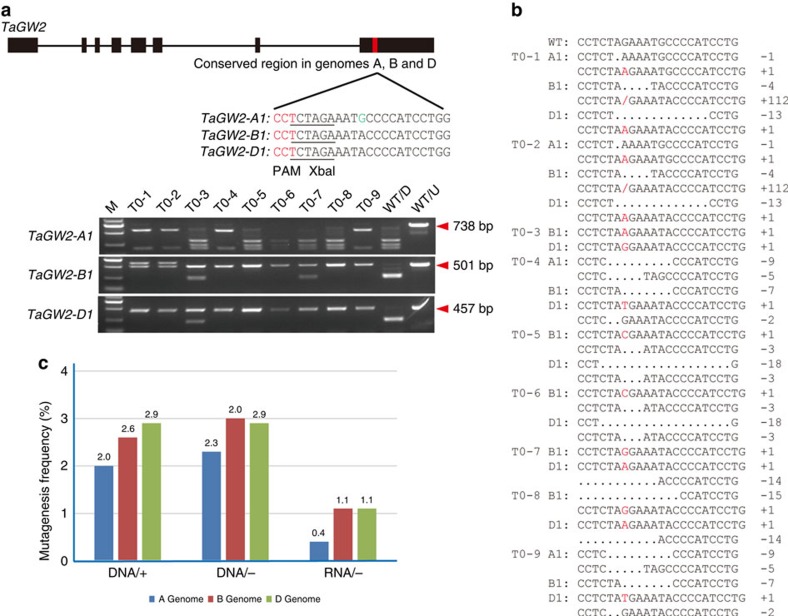
Development and testing of TECCRNA method. (**a**) Sites within a conserved region of exon 8 of wheat *TaGW2* homoeologues targeted by CRISPR/Cas9 systems. The PAM sequence is highlighted in red, the XbaI restriction sites are underlined and the single-nucleotide polymorphism in the targeted sequences is highlighted in green. Outcome of PCR-RE assays analysing nine representative *tagw2* mutants is shown. Lanes T0-1 to T0-9 show blots of PCR fragments amplified from independent wheat plants digested with XbaI. Lanes labelled WT/D and WT/U are PCR fragments amplified from WT plants with and without XbaI digestion, respectively. The bands marked by red arrowheads are caused by CRISPR/Cas9-induced mutations. (**b**) CRISPR/Cas9-induced mutant *TaGW2* alleles identified by sequencing. (**c**) Comparison the mutagenesis frequencies for three homoeologues of *TaGW2* of three genome-editing methods.

Although the chance of obtaining mutants with no detectable transgenes using the above method was high, a sizable proportion of the mutants still carried CRISPR/Cas9 DNA. Moreover, there is a possibility that the degradation products of CRISPR/Cas9 DNA may become integrated in the genome, which is difficult to be detected using PCR. Therefore, the genome-editing protocol was further optimized by transiently expressing the IVTs of Cas9-coding sequence and sgRNA in wheat callus cells (Fig. 1). The *TaGW2* gene, which acts as a negative regulator of kernel width and weight in bread wheat^14^, was used to test this transient expression of CRISPR/Cas9 RNA (abbreviated as TECCRNA)-based genome-editing method. The target site of the *TaGW2* gene was located in a conserved region in exon 8 (Fig. 3a, Supplementary Fig. 1 and Supplementary Table 1). We delivered Cas9 and sgRNA IVTs into the immature embryos of Kenong199 by particle bombardment, and regenerated the plants without herbicide selection as above (Fig. 1). From 1,600 bombarded immature embryos, 17 T0 mutants (efficiency 1.1%) were identified, among which 6 mutants (35.3%) contained site-specific indels in all six *TaGW2* alleles (Fig. 3a, Table 1 and Supplementary Table 2). These site-specific indels were confirmed by Sanger sequencing (Fig. 3b). To the best of our knowledge, this is the first report of producing genome-edited plants with CRISPR/Cas9 IVTs. It is well known that, under normal growth conditions, RNA molecules are unlikely to become integrated into nuclear DNA in plant cells. Therefore, we believe that the mutants produced with CRISPR/Cas9 IVTs are free of external nucleic acid integration. However, we noticed that the mutagenesis frequency of TECCRNA-based genome editing (1.1%) was somewhat lower than that obtained with TECCDNA (3.3%) or conventional DNA integration-based genome-editing methods (3.0%) in a side-by-side experiment (Table 1). The relatively low mutagenesis frequency of the TECCRNA method may be because RNA was less stable and could be easily degraded compared with DNA.

### Comparative analysis of off-target effects

Off-target is a main concern in current genome-editing studies. Thus, we compared potential off-target effects among the three wheat genome-editing methods outlined above. We computationally predicted the genome-wide potential off-target sites for TaGW2-sgRNA using the CasOT tool in bread wheat^15^. Eight likely off-target sites with three to four nucleotide mismatches to the recognition site of TaGW2*-*sgRNA were identified (Supplementary Table 4). However, none of these eight sites was mutated among the 67 *tagw2* mutants (24 by conventional DNA integration-based method, 26 by TECCDNA method and 17 by TECCRNA method) detected using PCR-RE assay. Similarly, we found in the bread wheat genome 24 potential off-target sites for TaGASR7-sgRNA with two to five nucleotide mismatches (Supplementary Table 4). Again, none of these 24 sites were disrupted in the 101 *tagasr7* mutants (including 80 in Bobwhite background and 21 in Kenong199 using the TECCDNA method).

Next, we examined off-target effects among highly similar bread wheat homoeologues using *TaGW2-A1, -B1* and *-D1* as example. The TaGW2*-*sgRNA recognition sequence was strictly conserved in *TaGW2-B1* and *TaGW2-D1*, but had one-nucleotide mismatch to the cognate target site in *TaGW2-A1* (Fig. 3a). We found that off-target frequencies caused by this one-nucleotide mismatch in *TaGW2-A1* were lower than on-target mutagenesis frequencies in *TaGW2-B1* and *-D1* using all three methods. The conventional DNA integration-based genome editing, TECCDNA and TECCRNA methods induced on-target mutations in *TaGW2-B1* (2.6%, 3.0% and 1.1%, respectively), *TaGW2-D1* (2.9%, 2.9% and 1.1%, respectively) and off-target mutations in *TaGW2-A1* (2.0%, 2.3% and 0.4%, respectively; Fig. 3c). The observed off-target effects may not be surprising because the one-nucleotide mismatch was located at position 9 of the protospacer-adjacent motif-proximal region of sgRNA, and many previous studies have found that mismatch around this position frequently leads to off-target mutagenesis^16^,^17^,^18^.

### Mutation transmission and phenotypic analysis

To investigate whether the mutations produced in this work can be transmitted to the next generation, representative transgene-free T0 *tagasr7, tadep1* and *talox2* mutants were self-pollinated, and their T1 progenies were analysed using PCR-RE. For homozygous mutations detected in T0 (including those with simultaneous editing of all six alleles), the transmission rates were 100%; for the majority of the heterozygous mutants, Mendelian segregation occurred (homozygote/heterozygote/wild type (WT): 1:2:1; Supplementary Table 5). As anticipated, all the T1 individuals were transgene-free (Supplementary Table 5).

**Figure 4:**
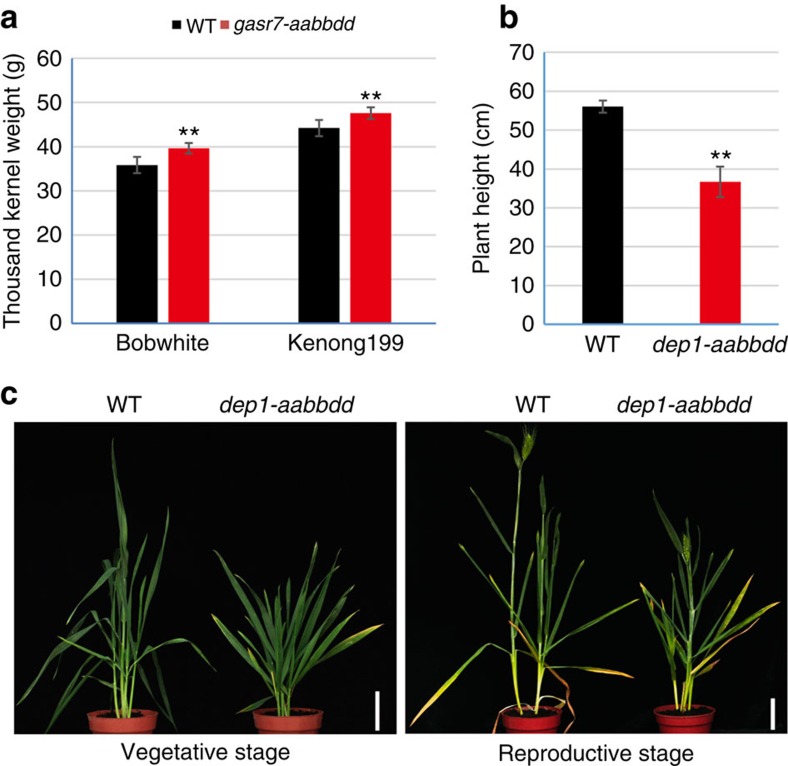
Phenotypic analysis of *TaGASR7* and *TaDEP1* mutants generated using TECCDNA genome-editing method. (**a**) TKW of WT and *tagasr7-aabbdd* mutant Bobwhite and Kenong199 plants. Data are from eight replicates for Bobwhite and ten replicates for Kenong199. (**b**) Plant heights of *tadep1-aabbdd* mutant plants compared with WT. Data are from 15 replicates. Values in **a**,**b** are mean±s.d. **P<0.01 (*t*-tests). (**c**) The morphology of plant heights of Kenong199 for WT and *tadep1-aabbdd* mutant in the vegetative and reproductive growth stages. Scale bar, 6 cm.

The phenotypic effects of the mutations in *TaGASR7* and *TaDEP1* were assessed. Homozygous, stable, transgene-free T2 mutants with all six alleles modified (that is, *aabbdd*) were identified and compared with WT controls. For *TaGASR7*, *aabbdd* mutant plants with frameshift mutations in all six alleles had significantly elevated thousand kernel weight (TKW), irrespective of the varietal background (*P*<0.01; Fig. 4a). This finding is consistent with the suggestion that *TaGASR7* is a negative regulator of grain weight in bread wheat^19^. In the case of *TaDEP1*, the *aabbdd* mutant plants with frameshift mutations in all six alleles exhibited an extremely dwarf phenotype (with a mean plant height of 36.7 cm) compared with their WT counterparts (mean plant height: 56.0 cm; *P*<0.01; Fig. 4b,c). These results demonstrate for the first time that *TaDEP1* is an important regulator of wheat growth and development.

## Discussion

Our work demonstrates that transient expression of CRISPR/Cas9 DNA or IVTs in wheat callus cells can efficiently induce targeted and transgene-free mutants. We successfully obtained homozygous mutants with no detectable transgenes for *TaGASR7*, *TaGW2* and *TaLOX2* in hexaploid bread wheat (cvs Bobwhite and Kenong199) and *TdGASR7* in tetraploid durum wheat (cvs Shimai11 and Yumai4) in the T0 generation. The mutations transmitted faithfully to the subsequent generations. These results suggest that our newly developed genome-editing methods are likely effective for both wheat species and are genotype-independent.

Production of specifically targeted, transgene-free mutants in the T0 generation through transient expression and function of CRISPR/Cas9 has received much attention in current plant genome-editing research because this type of approach minimizes the chance of off-target mutagenesis^20^,^21^,^22^, and the transgene-free mutants obtained in such a manner are likely exempt of transgene regulations. Furthermore, mutants lacking active transgenes obtained in the T0 generation can avoid the labourious, time-consuming crossing procedure to segregate away the CRISPR/Cas9 cassettes, which is specially difficult or even impossible for perennial plants and vegetatively propagated crops. Although it has been elegantly shown that transient expression of the CRISPR/Cas9 ribonucleoprotein complex in protoplasts can result in the production of specifically targeted, transgene-free mutants in the T0 generation in several plant species^6^, this method is unlikely efficient in major cereal crops (such as wheat, maize, rice and sorghum) as the regeneration from protoplasts has been reported to be very difficult^23^. Therefore, we develop two new, complementary genome-editing methods based on TECCDNA or TECCRNA in wheat callus cells. It is known that plant transformation consists of two consecutive steps—introduction of plasmid DNA into plant cells (transient transformation) and integration of introduced DNA into plant genome (stable transformation). As long as the plasmid is being introduced into plant cells, mutations could be generated no matter whether the CRISPR construct is integrated or not. The mutagenesis frequency of conventional DNA integration-based genome-editing method depends on plasmid integration. Thus, theoretically, the mutagenesis frequency of our TECCDNA genome-editing method may be higher than that of conventional DNA integration-based method. Judging from the data gathered from editing six genes in two wheat crop species, the mutagenesis frequencies can be as high as 9.5%, with up to 100% of the mutants in the T0 generation containing no detectable transgene. The high mutagenesis frequencies may also have been achieved because of the high activities of sgRNA optimized by the protoplast assays (Supplementary Fig. 1) and the efficient DNA transient delivery system, the latter of which was confirmed by the transient bombardment of immature embryos with the *gus* gene (Supplementary Fig. 6). Because callus cells are easy to induce and culture, and regeneration of plantlets from callus tissues can occur for many plant species with relative ease, we believe that the two methods described here should be broadly applicable in plant genome-editing studies.

Of the two methods developed, the one based on TECCDNA has a comparatively higher mutagenesis frequency, and is easier to perform because it omits the preparation of IVTs. However, many of the T0 mutants using TECCDNA are not transgene-free, and further efforts are needed to distinguish transgene-free mutants from those carrying transgenes. On the other hand, the TECCRNA method has a comparatively lower mutagenesis frequency, and is more difficult to accomplish because it requires the preparation and use of IVTs. However, none of the T0 mutants produced using TECCRNA have detectable transgenes and, given that no stable integration is likely to result from introduction of IVTs, no additional efforts are likely required to single out transgene-free mutants. Therefore, we suggest that the TECCDNA method may be useful for developing and optimizing a genome-editing protocol with callus cells as regeneration materials for a given plant species, and the mutants obtained are used for basic research (for example, functional validation of plant genes). After the genome-editing protocol is optimized, and gene function is validated, the TECCRNA method may then be more appropriate to produce transgene-free mutants for product development and potential commercialization.

The potential off-target effect is a major limitation for the wide application of the CRISPR/Cas9 system. Many studies have reported the off-target effects of the CIRSPR/Cas9 system in various organisms. For example, off-target effects occur at high frequencies in human cells^16^,^17^,^18^ and low frequencies in rice and soybean^24^,^25^. In our experiment examining potential off-target effects in the mutants obtained for *TaGASR7* or *TaGW2*, we did not find any off-targeting of the 32 sites that contain two to five nucleotide mismatches to the relevant sgRNA, suggesting that our TECCDNA and TECCRNA methods are highly specific in generating targeted mutations in wheat. We did observe off-target mutagenesis between the highly similar homoeologues of *TaGW2* because of strong conservation of their nucleotide sequence. However, this problem can be overcome through carefully designing copy-specific unique sgRNA in future research. Furthermore, the risk of off-targets is not as critical for plant research as clinical research because off-target mutations can be segregated away by crossing and only specific mutants will be chosen for basic research and plant breeding.

In summary, we developed two new and efficient genome-editing methods based on transient expression of CRISPR/Cas9 DNA or IVTs in wheat callus cells and regeneration of plants without herbicide or antibiotic selection. The effectiveness of the two methods in yielding specifically targeted mutants in the T0 generation with no detectable transgene was validated using six different wheat genes. Our methods are also likely useful for many other plant species that are hard-to-transform or regenerate from protoplasts, and for modifying the genes in vegetatively propagated crops such as potato, cassava and banana for which it is difficult to segregate away transgenes through selfing. We believe that the two methods described here, especially that based on TECCRNA, will accelerate molecular breeding studies in wheat, and help to stimulate more intensive genome-engineering research in plants.

## Methods

### Selection of sgRNA targets

Several sgRNA targets for each gene were designed in the conserved domains of the A, B and D genomes of wheat. The activities of the sgRNAs were evaluated by transforming the pJIT163-Ubi-Cas9 (ref. 9) and TaU6-sgRNA plasmids^26^ into wheat protoplasts. Total genomic DNA was extracted from the transformed protoplasts and fragments surrounding the targeted sequences were amplified with PCR. The PCR-RE assay was used to detect sgRNA activity^8^ (Supplementary Fig. 1).

### Protoplast assays

We used spring wheat variety Bobwhite and winter wheat variety Kenong199 in this study. Wheat protoplast transformation was performed according to ref. 8. In brief, the wheat seeds were grown at 25 °C for ∼6–10 days, and the protoplasts were isolated from wheat seedlings with the cell wall-dissolving enzyme solution. Polyethylene glycol 4000 (PEG4000)-mediated transformation was used to transform the plasmids into wheat protoplasts. After 48 h, the protoplasts were collected to extract DNA for PCR-RE assay.

### Construction of pGE-sgRNA vectors

Fragments of active TaU6-sgRNA (Supplementary Table 1) were amplified from the TaU6-sgRNA plasmid^26^ using the primer set U6-SpeI-F/sgRNA-SpeI-R with a SpeI restriction site (Supplementary Table 2). The PCR products were digested with SpeI and inserted into SpeI-digested pJIT163-Ubi-Cas9 (ref. 9) to yield the fused expression vector pGE-sgRNA (Fig. 2c).

### Mutant identification using the pooling method

Because the wheat plants were regenerated from callus without herbicide selection, many plantlets were obtained in our TECCDNA and TECCRNA assays. In order to save labour, we combined the plantlets regenerated from the same immature embryos as a pool (each pool usually contained three to four plantlets) to detect the mutations using PCR-RE assay. All the plantlets in the pools that gave positive PCR-RE signals were tested one by one with PCR-RE assay and subsequent sequencing.

### Biolistic delivery of TECCDNA

Plasmid DNA (pGE-sgRNA; Fig. 2c) was used to bombard wheat embryos. Biolistic bombardment was performed using a PDS1000/He particle bombardment system (Bio-Rad) with a target distance of 6.0 cm from the stopping plate at helium pressure 1,100 p.s.i. (ref. 27). After bombardment, embryos were transferred to callus induction medium. In the third week, all calli were transferred to regeneration medium. After 3–5 weeks, sprouts appeared on the surface of the calli. These were transferred to a rooting medium, and a large number of T0 seedlings were obtained about 1 week later. No selective agents were used in any part of the tissue culture process (Fig. 1).

### Southern blot analysis

Genomic DNA of wheat was isolated with cetyl trimethyl ammonium bromide buffer. About 35 μg of total DNA was digested by BamHI for 36 h. Then, the enzyme-digested products were separated using electrophoresis for 14 h in 0.8% agarose gels and transferred to a Hybond N^+^ membrane. Two fragments were amplified from pGE-TaGASR7 plasmid using primer sets described in Supplementary Table 2, and they were then used to generate [α-^32^P]dCTP-labelled probes using a Prime-a-Gene labelling system. Integrated plasmids were detected using a PhosphorImager.

### *In vitro* transcription of RNA

The 5′- and 3′-untranslated repeats of the *ZmUbi1* gene were cloned into pEasy-Blunt, which contains a T7 promoter, named as pLZT7. Full-length Cas9 cDNA flanked by two nuclear localization signal (NLS) (KRPAATKKAGQAKKKK) was cloned into the pLZT7 and linearized by XbaI. Capped Cas9 mRNA was transcribed using the Ambion mMESSAGE mMACHINE kit (AM1344). The templates for the synthesis of TaGW2-sgRNA were amplified from the pGE-TaGW2 plasmid using the primer set T7-GW2-F/sgRNA-PCR-R (Supplementary Table 2) and were purified with the PCR Purification Kit. TaGW2-sgRNA was synthesized using HiScribe T7 *In Vitro* Transcription Kit (E2030S).

### Biolistic delivery of TECCRNA

Cas9 mRNA (1 μg) and TaGW2-sgRNA (1 μg) were co-coated on the gold nanoparticle (0.6 μm) by adding 1/10 volume of 5 M ammonium acetate and 2 volumes of 2-propanol. The mixture was gently mixed by pipetting and was placed at −20 °C for at least 1 h. Biolistic bombardment was performed as in the TECCDNA method. After bombardment, the tissue culture process was performed in the same way as for the TECCDNA method.

### Conventional DNA integration-based genome editing

Plasmid DNAs (pGE-sgRNA (Fig. 2c) and pAHC20 (ref. 28)) were mixed in a 1:1 molar ratio before bombardment. We co-bombarded the immature embryos with pGE-TaGW2 plasmid and pAHC20, a plasmid harbouring the selectable *bar* gene. Biolistic bombardment was performed as in the TECCDNA method. After bombardment, embryos were transferred to the callus induction medium and developed for 2–4 weeks. Then, all calli were transferred to a selective regeneration medium for 4 weeks and selective rooting medium for another 4 weeks, supplemented with 5 mg l^−1^ phosphinothricin. After 10–12 weeks, T0 transgenic plants were obtained and used for mutagenesis detection.

### Phenotypic analysis of mutants

Plants were grown in a standard wheat field in 10 × 10 cm arrays under conventional cultivation conditions. Important agronomic traits including TKW and plant height were measured on a single-plant basis. About 100 filled grains of Bobwhite and about 150 filled grains of Kenong199 were picked for TKW measurements because winter wheat usually has more seeds than spring wheat.

### Data availability

The authors declare that all data supporting the findings of this study are available within the article and its Supplementary Information files or are available from the corresponding author upon request.

## Additional information

**How to cite this article:** Zhang, Y. *et al*. Efficient and transgene-free genome editing in wheat through transient expression of CRISPR/Cas9 DNA or RNA. *Nat. Commun.* 7:12617 doi: 10.1038/ncomms12617 (2016).

## Supplementary Material

Supplementary Information Supplementary Figures 1 - 6 and Supplementary Tables 1 - 5

## Figures and Tables

**Table 1:** The mutagenesis frequencies of three methods for wheat genome editing in T0 generation.

**Gene**	**Wheat variety**	**Delivery form/Selection (+/−)**	**No. of bombarded embryos**	No. of mutants/mutagenesis (%)*	**No. of homozygous mutants/frequency (%)**†	**No. of transgene-free mutants/frequency (%)**‡	**No. of homozygous transgene-free mutants/frequency (%)**§
*TaGASR7*	Bobwhite	DNA/−	1,600	80 (5.0)	8 (10.0)	35 (43.8)	4 (5.0)
	Kenong199	DNA/−	800	21 (2.6)	4 (19.0)	13 (61.9)	1(4.8)
*TaGW2*	Kenong199	DNA/+	800	24 (3.0)	9 (37.5)	0	0
	Kenong199	DNA/−	800	26 (3.3)	7 (26.9)	16 (61.5)	2 (7.7)
	Kenong199	RNA/−	1,600	17 (1.1)	6 (35.3)	17 (100)	6 (35.3)
*TaDEP1*	Kenong199	DNA/−	1,280	26 (2.0)	0	14 (53.8)	0
*TaNAC2*	Kenong199	DNA/−	800	16 (2.0)	N.D.	12 (75.0)	N.D.
*TaPIN1*	Kenong199	DNA/−	800	8 (1.0)	N.D.	5 (62.5)	N.D.
*TaLOX2*	Kenong199	DNA/−	800	76 (9.5)	34 (44.7)	66 (86.8)	28 (36.8)
*TdGASR7*	Shimai11	DNA/−	800	11 (1.4)	3 (27.3)	6 (54.5)	0
	Yumai4	DNA/−	800	12 (1.5)	5 (41.7)	7 (58.3)	2 (16.7)

CRISPR, clustered regularly interspaced short palindromic repeat; N.D., not detected.

^*^On the basis of the number of plants carrying the observed mutations over the total number of bombarded immature embryos.

^†^On the basis of the number of plants carrying mutations on all the six alleles over the total number of mutant plants.

^‡^On the basis of the number of mutant plants not harbouring CRISPR DNA construct over the total number of mutant plants tested.

^§^On the basis of the number of plants carrying mutations on all the six alleles and not harbouring CRISPR DNA construct over the total number of mutant plants. ‘+' with herbicide selection; ‘−' without herbicide selection.
