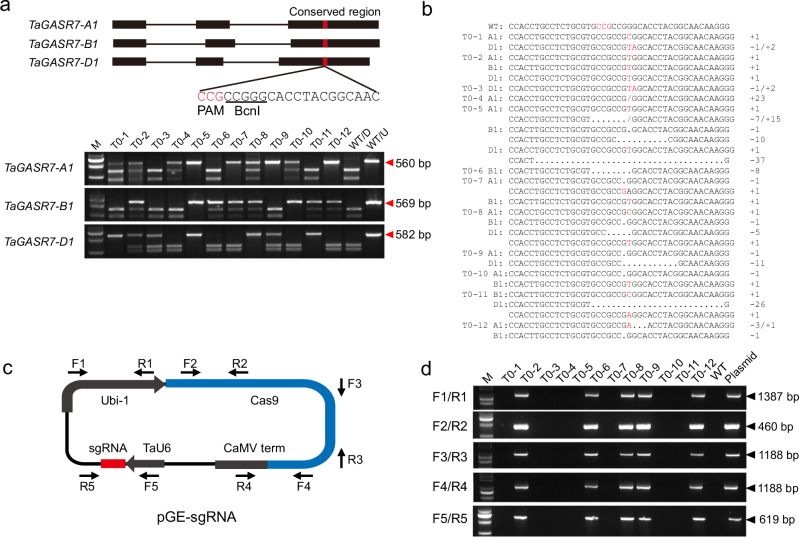# Publisher Correction: Efficient and transgene-free genome editing in wheat through transient expression of CRISPR/Cas9 DNA or RNA

**DOI:** 10.1038/s41467-025-59702-5

**Published:** 2025-05-09

**Authors:** Yi Zhang, Zhen Liang, Yuan Zong, Yanpeng Wang, Jinxing Liu, Kunling Chen, Jin-Long Qiu, Caixia Gao

**Affiliations:** 1 State Key Laboratory of Plant Cell and Chromosome Engineering, Institute of Genetics and Developmental Biology, Chinese Academy of Sciences, Beijing, 100101 China; 2 University of Chinese Academy of Sciences, Beijing, 100049 China; 3 State Key Laboratory of Plant Genomics, Institute of Microbiology, Chinese Academy of Sciences, Beijing, 100101 China

Correction to: *Nature Communications* 10.1038/ncomms12617, published online 25 August 2016

In the version of the article initially published, in the top panel of Fig. 2a, the lower label “*TaGASR7-B1*” should have read “*TaGASR7-D1*”, as shown in Fig. 2 below. This notice serves to correct the figure.

Fig. 2 Corrected