# Supplementary material for: Efficient and transgene-free genome editing in wheat through transient expression of CRISPR/Cas9 DNA or RNA
Source: Nat Commun. 2016 Aug 25;7:12617. doi: 10.1038/ncomms12617 (PMC5007326; doi:10.1038/ncomms12617)
Supplement: Supplementary Information — Supplementary Figures 1 - 6 and Supplementary Tables 1 - 5 [file ncomms12617-s1.pdf]

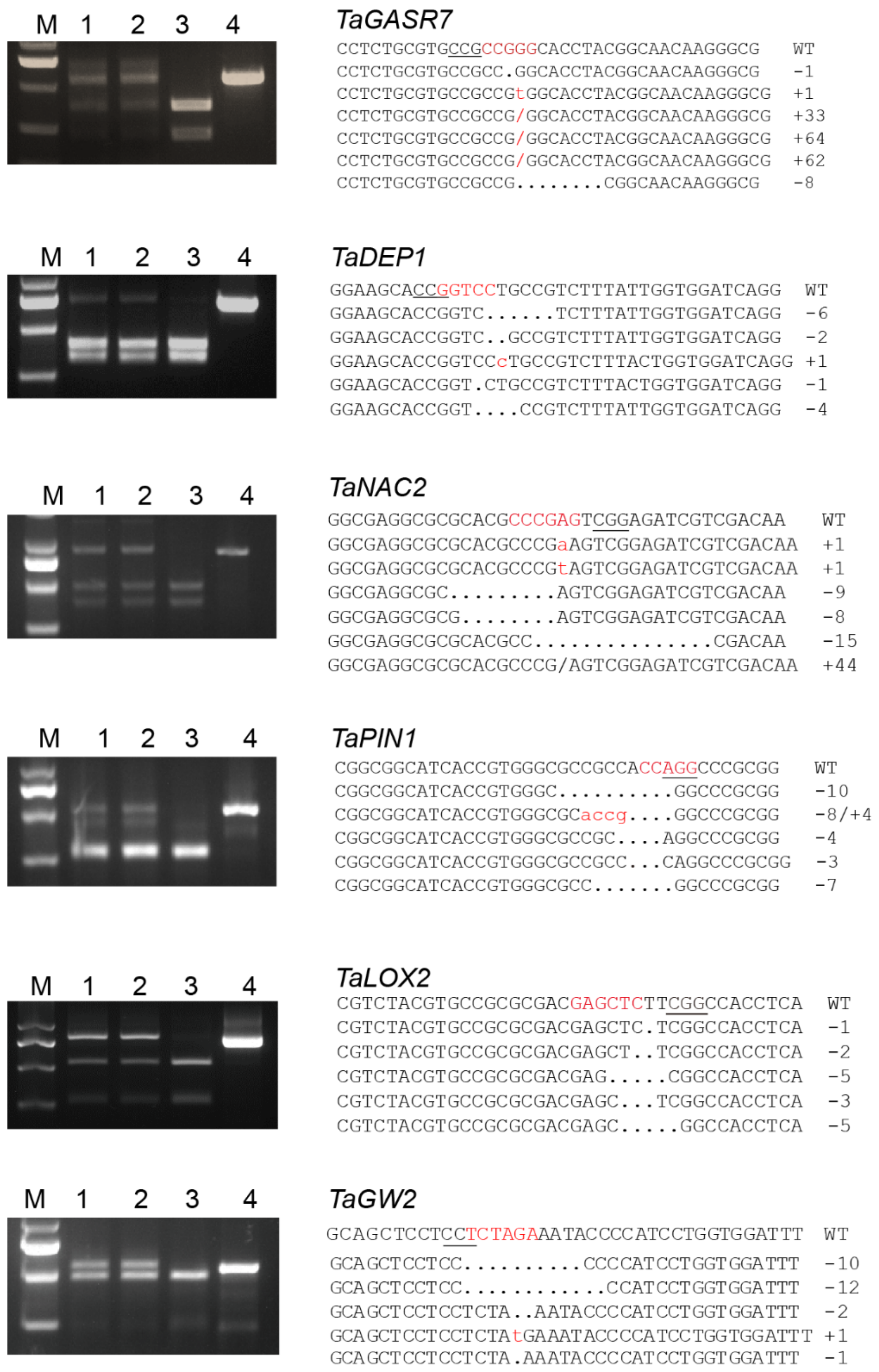

Supplementary Figure 1. CRISPR/Cas9-induced targeted mutations in *TaGASR7*, *TaDEP1*, *TaNAC2*, *TaPIN1*, *TaLOX2* and *TaGW2* genes in wheat protoplasts. Lanes 1 and 2: digested CRISPR/Cas9-transformed protoplasts; lanes 3 and 4: digested and undigested wild type controls; M: marker. Sequences of CRISPR/Cas9-induced mutations are shown on the right. The wild type sequences are shown at the top of each sequence group. The numbers at the sides indicate the type of mutations and how many nucleotides are involved.

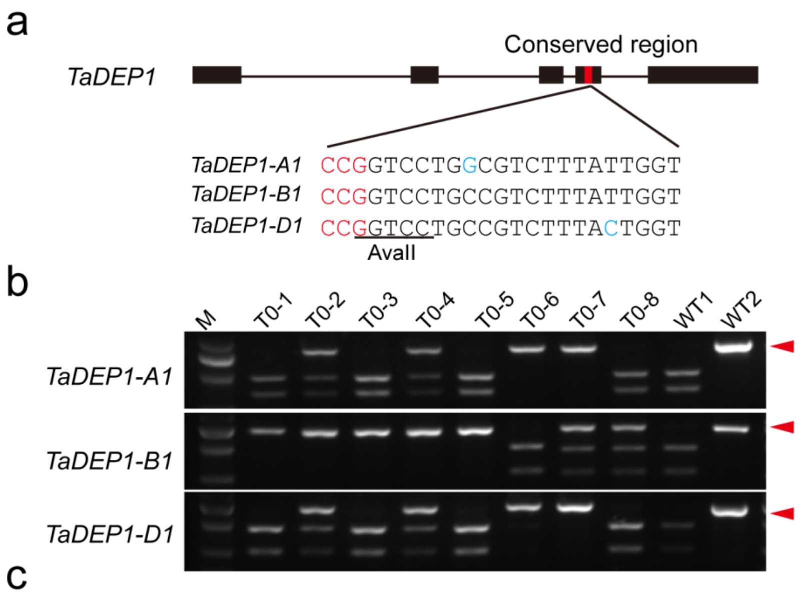

c

```
WT:         AAGCACCGGTCCTGCCGTCTTTATTGGTGGATCA
T0-1 B1:    AAGCACCGGTCtCTGCCGTCTTTATTGGTGGATCA   +1
T0-2 A1:    AAGCACCGGTC..GGCGTCTTTATTGGTGGATCA    -2
     B1:    AAGCACCGGTCaCTGCCGTCTTTATTGGTGGATCA   +1
     D1:    AAGCACCGGTCcCTGCCGTCTTTACTGGTGGATCA   +1
            AAGCACCGGT.CTGCCGTCTTTACTGGTGGATCA    -1
T0-3 B1:    AAGCACCGGTCaCTGCCGTCTTTATTGGTGGATCA   +1
T0-4 A1:    AAGCACCGGTC..GGCGTCTTTATTGGTGGATCA    -2
     B1:    AAGCACCGGTCaCTGCCGTCTTTATTGGTGGATCA   +1
            AAGCACCGG......................TCA   -22
     D1:    AAGCACCGGTCcCTGCCGTCTTTACTGGTGGATCA   +1
            AAGCACCGGT.CTGCCGTCTTTACTGGTGGATCA    -1
T0-5 B1:    AAGCACCGGTC.TGCCGTCTTTATTGGTGGATCA    -1
            AAGCACCGGTC..GCCGTCTTTATTGGTGGATCA    -2
T0-6 A1:    AAGCACCGGTCaCTGGCGTCTTTATTGGTGGATCA   +1
     D1:    AAGCACCGGTC..GCCGTCTTTACTGGTGGATCA    -2
T0-7 A1:    AAGCACCGGTC.TGGCGTCTTTATTGGTGGATCA    -1
            AAGCACCGGTCaCTGGCGTCTTTATTGGTGGATCA   +1
     B1:    AAGCACCGGTCtCTGCCGTCTTTATTGGTGGATCA   +1
     D1:    AAGCACCGGT.CTGCCGTCTTTACTGGTGGATCA    -1
T0-8 B1:    AAGCACCGGT.CTGCCGTCTTTATTGGTGGATCA    -1
```

Supplementary Figure 2. Outcome of PCR-RE assays for 8 representative *tadep1* mutants. (a) Sites within a conserved region of exon 4 of *TaDEP1* homoeologs targeted by CRISPR/Cas9. Of the two SNPs highlighted in blue. (b) Lanes T0-1 to T0-8 show PCR fragments amplified from independent regenerated wheat plants digested with *AvaII*. Lanes labeled WT1 and WT2 show PCR fragments amplified from a wild type plant with and without *AvaII* digestion, respectively. The bands marked by red arrowheads result from CRISPR/Cas9-induced mutations. (c) Genotypes of 8 representative mutant plants identified by sequencing. "–" and "+" indicate deletion and insertion of the given number of nucleotides, respectively.

a

TaNAC2

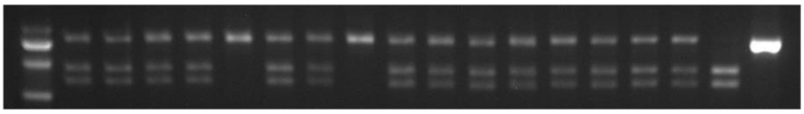

```
       GGCGAGGCGCGCACGCCCGAGTCGGAGATCGTCGACAA    WT
T0-1:  GGCGAGGCGCGCACGCCCG/AGTCGGAGATCGTCGACAA   +5
T0-2:  GGCGAGGCGCGCACGCCCGaAGTCGGAGATCGTCGACAA   +1
T0-3:  GGCGAGGCGCGCACGC...AGTCGGAGATCGTCGACAA    -3
T0-4:  GGCGAGGCGCGCACGCCCGtAGTCGGAGATCGTCGACAA   +1
T0-5:  GGCGAGGCGCGCACGCCC..GTCGGAGATCGTCGACAA    -2
       GGCGAGGCGCGCACGCCCGaAGTCGGAGATCGTCGACAA   +1
```

b

TaPIN1

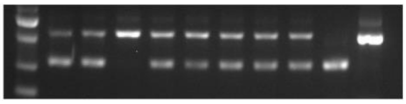

```
       CGGCGGCATCACCGTGGGCGCCGCCACCAGGCCCGCGG    WT
T0-1:  CGGCGGCATCACCGTGGGCGCCG.....AGGCCCGCGG    -5
       CGGCGGCATCACCGTGGGC.........AGGCCCGCGG    -9
T0-2:  CGGCGGCATCACCGTGGGCGCCG....CAGGCCCGCGG    -4
T0-3:  CGGCGGCATCACCGTGGGCG.......CAGGCCCGCGG    -7
       CGGCGGCATCACCGTGGGCGCCG....CAGGCCCGCGG    -4
T0-4:  CGGCGGCATCACCGTGGGCGCCGCC....GGCCCGCGG    -4
       CGGCGGCATCACCGTGGGCGCC.......GGCCCGCGG    -7
```

c

TaLOX2

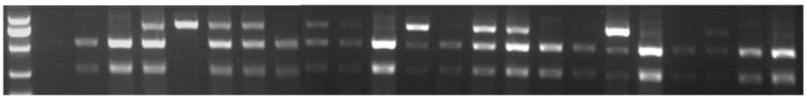

```
       AACGTCTACGTGCCGCGCGACGAGCTCTTCGGCCACCT    WT
T0-1:  AACGTCTACGTGCCGCGCGACG....CTTCGGCCACCT    -4
T0-2:  AACGTCTACGTGCCGCGCGACGAG....TCGGCCACCT    -4
       AACGTCTACGTGCCGCGCGACGAGC....CGGCCACCT    -4
T0-3:  AACGTCTACGTGCCGCGCGACGAGC.CTTCGGCCACCT    -1
T0-4:  AACGTCTACGTGCCGCGCGACGA...CTTCGGCCACCT    -3
T0-5:  AACGTCTACGTGCCGCGC.....GCTCTTCGGCCACCT    -5
T0-6:  AACGTCTACGTGCCGCG.......CTCTTCGGCCACCT    -7
T0-7:  AACGTCTACG.............GCTCTTCGGCCACCT    -13
```

Supplementary Figure 3. Outcome of PCR-RE assays for *tanac2* (a), *tapin1* (b) and *talox2* (c) mutants.

a

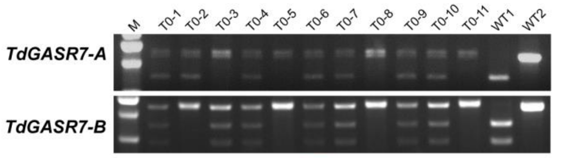


```
      WT: CCACCTGCCTCTGCGTGCCGCCGGGCACCTACGGCAACAAGGG
T0-1 A1: CCACCTGCCTCTGCGTGCCGCCG/GGCACCTACGGCAACAAGGG   +2
     B1: CCACCTGCCTCTGCGTGCCGCCG..GCACCTACGGCAACAAGGG   -2
T0-2 A1: CCACCTGCCTCTGCGTGCCGCCGTGGCACCTACGGCAACAAGGG   +1
     B1: CCACTTGCCTCTGCGTGCCGCCG...CACCTACGGCAACAAGGG   -3
         CCACCTGCCTCTGCGTGCCGCCGTGGCACCTACGGCAACAAGGG   +1
T0-3 A1: CCACCTGCCTCTGCGTGCCGCCGTAGCACCTACGGCAACAAGGG   -1/+2
         CCACCTGCCTCTGCGTGCCGC....GCACCTACGGCAACAAGGG   -4
     B1: CCACCTGCCTCTGCGTGCCGC......ACCTACGGCAACAAGGG   -6
T0-4 A1: CCACCTGCCTCTGCGTGCCGCCG/GGCACCTACGGCAACAAGGG   +12
     B1: CCACCTGCCTCTGCGTGCCGCCGTGGCACCTACGGCAACAAGGG   +1
T0-5 A1: CCACCTGCCTCTGCGTGCCGCCGTGGCACCTACGGCAACAAGGG   +1
     B1: CCACTTGCCTCTGCGTGCCGCCG.GCACCTACGGCAACAAGGG    -1
         CCACTTGCCTCTGCGTGCCGC..........CGGCAACAAGGG    -10
T0-6 A1: CCACCTGCCTCTGCGTGCC.....GCACCTACGGCAACAAGGG    -5
     B1: CCACTTGCCTCTGCGT........GCACCTACGGCAACAAGGG    -8
T0-7 A1: CCACCTGCCTCTGCGTGCCGCC.GGCACCTACGGCAACAAGGG    -1
     B1: CCACTTGCCTCTGCGTGCCGCCGTGGCACCTACGGCAACAAGGG   +1
T0-8 A1: CCACCTGCCTCTGCGTGCCGCCG/GGCACCTACGGCAACAAGGG   +7
     B1: CCACTTGCCTCTGCGTGCCGCC.GGCACCTACGGCAACAAGGG    -1
         CCACCTGCCTCTGCGTGCC......CACCTACGGCAACAAGGG    -6
T0-9 A1: CCACCTGCCTCTGCGTGCCGCC.GGCACCTACGGCAACAAGGG    -1
     B1: CCACCTGCCTCTGCGTGCCG.............GCAACAAGGG   -13
T0-10 A1:CCACCTGCCTCTGCGTGCCGCC.GGCACCTACGGCAACAAGGG    -1
      B1:CCACTTGCCTCTGCGTGCCGCCGTGGCACCTACGGCAACAAGGG   +1
T0-11 A1:CCACTTGCCTCTGCGTGCCGCCGCGGCACCTACGGCAACAAGGG   +1
         CCACTTGCCTCTGCGTGCCGCCGTGGCACCTACGGCAACAAGGG   +1
      B1:CCACTTGCCTCTGCGTGCCGCC.GGCACCTACGGCAACAAGGG    -1
         CCACCTGCCTCTGCGTGCCGCCGAGGCACCTACGGCAACAAGGG   +1
```

b

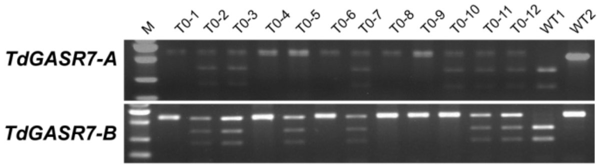


```
      WT: CCACCTGCCTCTGCGTGCCGCCGGGCACCTACGGCAACAAGGG
T0-1 A1: CCACCTGCCTCTGCGTGCCGCCG/GGCACCTACGGCAACAAGGG   +5
         CCACTTGCCTCTGCGTGCCGCC.GGCACCTACGGCAACAAGGG    -1
     B1: CCACCTGCCTCTGCGTGCCGCC...CACCTACGGCAACAAGGG    -3
         CCACTTGCCTCTGCGTGCCGCCG.GCACCTACGGCAACAAGGG    -1
T0-2 A1: CCACCTGCCTCTGCGTGCCGCCGTGGCACCTACGGCAACAAGGG   +1
     B1: CCACCTGCCTCTGCGTGCCGCCGAGGCACCTACGGCAACAAGGG   +1
T0-3 A1: CCACCTGCCTCTGCGTGCCGCCG/GGCACCTACGGCAACAAGGG   +2
     B1: CCACCTGCCTCTGCGTGCCGC....CACCTACGGCAACAAGGG    -4
T0-4 A1: CCACCTGCCTCTGCGTGCCGCCG/GGCACCTACGGCAACAAGGG   +12
         CCACCTGCCTCTGCGTGCCGC...GCACCTACGGCAACAAGGG    -3
     B1: CCACCTGCCTCTGCGTGCCGCCGAGGCACCTACGGCAACAAGGG   +1
T0-5 A1: CCACCTGCCTCTGCGTGCCGCCG/GGCACCTACGGCAACAAGGG   +2
         CCACTTGCCTCTGCGTGCCGC.../GCACCTACGGCAACAAGGG   -3/+7
     B1: CCACTTGCCTCTGCGTGCCGCCG........CGGCAACAAGGG    -8
T0-6 A1: CCACCTGCCTCTGCGTGCC.....GCACCTACGGCAACAAGGG    -5
         CCACCTGCCTCTGCG.........GCACCTACGGCAACAAGGG    -9
     B1: CCACTTGCCTCTGCGT........GCACCTACGGCAACAAGGG    -8
         CCACTTGCCTCTGCGTGCCGCCGTGGCACCTACGGCAACAAGGG   +1
T0-7 A1: CCACCTGCCTCTGCGTGCCGCC.GGCACCTACGGCAACAAGGG    -1
     B1: CCACTTGCCTCTGCGTGCCGCCG/GGCACCTACGGCAACAAGGG   +6
T0-8 A1: CCACCTGCCTCTGCGTGCCGCCGAGGCACCTACGGCAACAAGGG   +1
     B1: CCACTTGCCTCTGCGTGCCGCC.GGCACCTACGGCAACAAGGG    -1
         CCACCTGCCTCTGCGTGCC......CACCTACGGCAACAAGGG    -6
T0-9 A1: CCACCTGCCTCTGCGTGCCGCC.GGCACCTACGGCAACAAGGG    -1
         CCACCTGCCTCTGCGTGCCGCCG/GGCACCTACGGCAACAAGGG   +4
     B1: CCACCTGCCTCTGCGTGCCGCCGAGGCACCTACGGCAACAAGGG   +1
         CCACCTGCCTCTGCGTGCCGCC...........GCAACAAGGG   -11
T0-10 A1:CCACCTGCCTCTGCGTGCCGCC.GGCACCTACGGCAACAAGGG    -1
      B1:CCACCTGCCTCTGCGTGCCGCCGAGGCACCTACGGCAACAAGGG   +1
         CCACTTGCCTCTGCGTGCCGCCG/GGCACCTACGGCAACAAGGG   +2
T0-11 A1:CCACTTGCCTCTGCGTGCCGCC.GGCACCTACGGCAACAAGGG    -1
      B1:CCACTTGCCTCTGCGTGCCGCC.GGCACCTACGGCAACAAGGG    -1
T0-12 A1:CCACTTGCCTCTGCGTGCCGCC....ACCTACGGCAACAAGGG    -1
      B1:CCACTTGCCTCTGCGTGCCGC...GCACCTACGGCAACAAGGG    -1
```

Supplementary Figure 4. Outcome of PCR-RE assays for tetraploid *tdgasr7* mutants in Shimai11 (a) and Yumai4 (b) with specific primers.

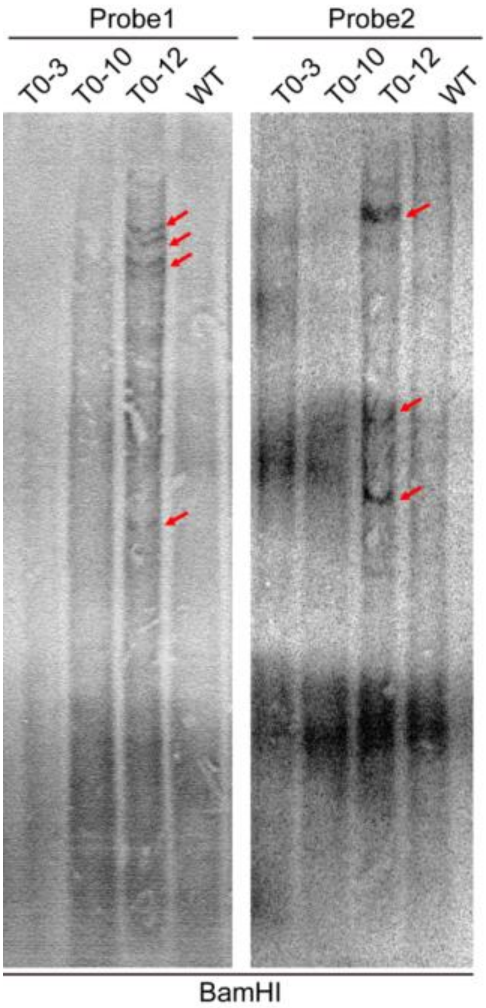

Supplementary Figure 5. Outcome of Southern blot analysis for 3 representative *tagasr7* mutant plants. Genomic DNA of wheat was isolated and digested with *BamHI*. The enzyme-digested products were transferred to a Hybond N^+ membrane and hybridized with two ^{32}P-labeled probes, probe1 and probe2. T0-3 and T0-10 are transgene-free mutants. T0-12 is a transgenic mutant. WT represents the wild type plant. The bands marked by red arrowheads represent the hybridizing signals in the transgenic mutant.

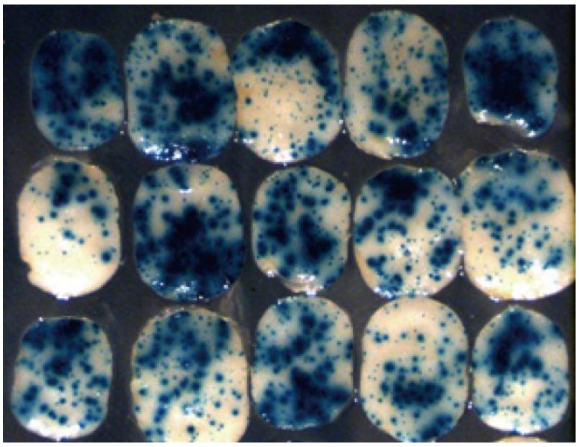

Supplementary Figure 6. Outcome of the transient bombardment of wheat immature embryos with *gus* gene. A large number of transfected cells confirmed the high efficiency of DNA transient delivery system.

Supplementary Table 1. SgRNA target loci and sequences.

Gene name	Target site	Oligo-F	Oligo-R	Detection method
sg-GASR7	CCGCCGGGCACCTACGGCAAC	CTTGGTTGCCGTAGGT GCCCGG	AAACCCGGGCACCTACGG CAAC	PCR/RE *BcnI*
sg-DEP1	CCGGTCCTGCCGTCTTTATTGGT	CTTGACCAATAAAGAC GGCAGGAC	AAACGTCCTGCCGTCTTT ATTGGT	PCR/RE *AvaII*
sg-NAC2	CGAGGCGCGCACGCCCGAGTCGG	CTTGCGAGGCGCGCAC GCCCGAGT	AAACACTCGGGCGTGCGC GCCTCG	PCR/RE *AvaI*
sg-PIN1	TCACCGTGGGCGCCGCCACCAGG	CTTGTCACCGTGGGCG CCGCCACC	AAACGGTGGCGGCGCCCA CGGTGA	PCR/RE *MvaI*
sg-LOX2	GTGCCGCGCGACGAGCTCTTCGG	CTTGTGCCGCGCGACG AGCTCTT	AAACAAGAGCTCGTCGCG CGGCA	PCR/RE *SacI*
sg-GW2	CCTCTAGAAATACCCCATCCTG	CTTGCAGGATGGGGTA TTTCTAG	AAACCTAGAAATACCCCA TCCTG	PCR/RE *XbaI*

Supplementary Table 2. PCR primers and their applications.

Primer name	Primer sequence	Application
F1<br>R1	CAGTTAGACATGGTCTAAAGGACAATTGAG<br>CCAACCACACCACATCATCACAACCAA	Detecting CRISPR construct
F2<br>R2	CCTAAGAAGAAGAGAAAGGTCG<br>GCAGATGATAGATTGTGGGGTA	Detecting CRISPR construct and labelling probe 1 for Southern blot
F3<br>R3	GCCCATCTCTTCGATGACAAGGTTATG<br>CTTCGCAGTGGCCTTGCCAATTTC	Detecting CRISPR construct
F4<br>R4	GGTGGCTTACTCTGTCCTGGTT<br>TTCCTTGTCTTCCTCCTTCCTT	Detecting CRISPR construct and labelling probe 2 for Southern blot
F5<br>R5	AGCCCGTTATTCTGACAGTTCTGGTGC<br>GTGAGCGCAACGCAATTAATGTGAG	Detecting CRISPR construct
U6-SpeI-F<br>sgRNA-SpeI-R	CGGACTAGTGACCAAGCCCGTTATTCTGAC<br>CGGACTAGTAAAAAAAGCACCGACTCGGTGCCAC	Amplifying the fragment of TaU6-sgRNA
GASR7-F<br>GASR7-R	GGAGGTGATGGGAGGTGGGGG<br>CTGGGAGGGCAATTCACATGCCA	Amplifying the *TaGASR7* target site
GASR7-A1/B1/D1-F	CCTTCATCCTTCAGCCATGCAT	Amplifying the *TaGASR7* target site
GASR7-A1-R	CCACTAAATGCCTATCACATACG	Amplifying the *TaGASR7-A1* target site
GASR7-B1-R	AGGGCAATTCACATGCCACTGAT	Amplifying the T*aGASR7-B1* target site
GASR7-D1-R	CCTCCATTTTTCCACATCTTAGTCC	Amplifying the *TaGASR7-D1* target site
DEP1-F<br>DEP1-R	CAGCAAACCCCACTTGCATCAGAAC<br>CAGCACAGCCATGAAGCACATATGCA	Amplifying the *TaDEP1* target site
DEP1-A1/B1/D1-F	CCAGCAAACCCCACTTGCATCAGAAC	Amplifying the *TaDEP1* target site
DEP1-A1-R	GTGTACTCTACTGGAAGGAAGGTTACAAG	Amplifying the *TaDEP1-A1* target site
DEP1-B1-R	CAGATGAATACTGCGTCCCTACGATAGGT	Amplifying the *TaDEP1-B1* target site
DEP1-D1-R	CTGGGCGGGTTCAGTTAGCAGGTTAC	Amplifying the *TaDEP1-D1* target site
NAC2-F<br>NAC2-R	GGGATCAAGAAGGCCCTGGTGTTT<br>TCGATCTCGGTATCTGACGGTCTGTG	Amplifying the *TaNAC2* target site
PIN1-F<br>PIN1-R	GATGGACTTCATGATGATCGCC<br>AACGGCACCGAGGCGTACAACGA	Amplifying the *TaPIN1* target site
LOX2-F<br>LOX2-R	CGTCTACCGCTACGACGTCTACAACG<br>GGTCGCCGTACTTGCTCGGATCAAGT	Amplifying the *TaLOX2* target site
GW2-F<br>GW2-R	ATGCCAACCCTTGCGTGTGCGT<br>TCCTGCTTGTGGGAGCTTTATG	Amplifying the *TaGW2* target site
GW2-A1-F	CTGCCATTACTTTGTATTTTGGTAATA	Amplifying the *TaGW2-A1* target site
GW2-B1-F	GTTCAGATGGCAATCTAAAAGTT	Amplifying the *TaGW2-B1* target site
GW2-D1-F	GCATGTACTTTGATTGTTTGCGTGA	Amplifying the T*aGW2-D1* target site
GW2-A1/B1/D1-R	TCCTTCCTCTCTTACCACTTCCC	Amplifying the *TaGW2* target site
T7-GW2-F<br>sgRNA-PCR-R	TAATACGACTCACTATAGGCAGGATGGGGTATTTCTAG<br>GCACCGACTCGGTGCCACTT	Amplifying the T7-GW2-sgRNA
ZmUbi-5’UTR-F<br>ZmUbi-KH-5’UTR-R	TTCCCCAACCTCGTGTTGTTCGGAG<br>ACGACCCATGACAAGCTTTAGCGGTACCCTTGAAGCGGAGGTGCCGACG	Amplifying the 5’UTR of *ZmUbi1* for pLZT7 cloning
ZmUbi-KH-3’UTR-F<br>ZmUbi-polyA-3’UTR-R	CTCCGCTTCAAGGGTACCGCTAAAGCTTGTCATGGGTCGTTTAAGCTGC<br>TCTAGATTTTTTTTTTTTTTTTTTTTTTTTTTTTTTTTTTTCGCACACACAACACAACCGGT	Amplifying the 3’UTR of *ZmUbi1* for pLZT7 cloning
KpnI-zCas9-F<br>HindIII-zCas9-R	GGGGGTACCATGGATTACAAGGACCACGACG<br>CCCAAGCTTTCACTTCTTCTTCTTCGCCTGC	Amplifying the KpnI-zCas9-HindIII for pLZT7-zCas9 cloning

Supplementary Table 3. Genotypes of the 80 T0 *tagasr7* mutants with respect to mutations in the *TaGASR7-A1*, *-B1* and *-D1* homoeologs.

Genotype of *TaGASR7* homoeologs	Plant ID	Mutation detected (bp)[a]	CRISPR -free[b]	Genotype of *TaGASR7* homoeologs	Plant ID	Mutation detected (bp)[a]	CRISPR -free[b]
AaBBDD	T0-4	+23(Aa)	YES	AaBbdd	T0-8	+1(Aa);-1(Bb); -5,+1(dd)	NO
	T0-28	N.D.	NO		T0-52	N.D.	YES
	T0-36	+12(Aa)	YES		T0-58	N.D.	NO
aaBBDD	N.A.				T0-66	N.D.	YES
AABbDD	T0-6	-8(Bb)	NO	AabbDd	T0-31	+1(Aa); -4/+1, +1(bb); +8(Dd)	YES
	T0-49	+123(Bb)	YES		T0-33	N.D.	NO
AAbbDD	N.A.				T0-50	N.D.	NO
AABBDd	T0-3	-1/+2(Dd)	YES		T0-55	N.D.	NO
	T0-30	N.D.	NO		T0-69	N.D.	YES
	T0-56	N.D.	NO		T0-76	N.D.	NO
	T0-79	+1(Dd)	YES		T0-78	N.D.	NO
AABBdd	T0-71	+63, -1(dd)	YES	Aabbdd	T0-16	-6/+1(Aa); -25(bb); +1,-26(dd)	YES
AaBbDD	T0-15	+82(Aa); -1(Bb)	NO		T0-23	-4(Aa); +1,+89(bb); -6,+1(dd)	NO
	T0-44	N.D.	YES		T0-38	N.D.	NO
	T0-60	N.D.	NO		T0-40	N.D.	NO
AabbDD	T0-10	-1(Aa); +1(bb)	YES		T0-45	N.D.	YES
	T0-14	-1(Aa);	NO		T0-68	N.D.	YES
aaBbDD	T0-7	-1, +1(aa); +1(Bb)	YES	aaBbDd	T0-19	+129,+1(aa);-2/+1(Bb);-16 (Dd)	NO
	T0-12	-3/+1(aa); -1(Bb)	NO		T0-32	N.D.	NO
AaBBDd	T0-35	+1(Aa); +1(Dd)	NO		T0-57	N.D.	NO
	T0-51		YES		T0-59	N.D.	NO
	T0-61	+2(Aa); -6(Dd)	YES		T0-64	N.D.	NO
	T0-77	N.D.	YES	aaBbdd	T0-17	-2,-3(aa); -1(Bb); -10,-7(dd)	NO
AaBBdd	T0-1	+1 (Aa); -1/+2 (dd)	YES		T0-26	N.D.	NO
aaBBDd	T0-9	-1(aa); -11(Dd)	NO		T0-41	+8,-1(aa);+24(Bb); +25,+1(dd)	YES
aaBBdd	N.A.				T0-54	N.D.	YES
aabbDD	N.A.				T0-62	N.D.	NO
AABbDd	T0-22	+1(Bb); -3(Dd)	NO		T0-70	N.D.	NO
	T0-24	-7(Bb); +82(Dd)	NO	aabbDd	T0-13	+1(aa); +1,-12(bb); -1(Dd)	NO
	T0-42	N.D.	YES		T0-37	+3,-1(aa); +1,-2/+1(bb); -2(Dd)	YES
	T0-46	N.D.	NO		T0-43	N.D.	YES
	T0-74	N.D.	NO		T0-63	N.D.	NO
AABbdd	T0-11	+1(Bb); -26, +1(dd)	YES		T0-80	N.D.	YES
AAbbDd	N.A.			AaBbDd	T0-2	+1(Aa); +1(Bb); +1(Dd)	NO
AAbbdd	N.A.				T0-18	-4/+1(Aa); +2(Bb); +1(Dd)	NO
aabbdd	T0-5	+1,-7/+15(aa); -1,-10(bb); +1,-37(dd)	YES		T0-20	-6(Aa); -17(Bb); +5(Dd)	YES
	T0-27	+1(aa); -1,-7(bb); -5,+1(dd)	YES		T0-21	-5/+1(Aa); +1(Bb); +1, -19/+166, -19/+167(Dd)	NO
	T0-29	-4/+1(aa); +63,+1(bb); +1,-1(dd)	YES		T0-25	+1(Aa); -9(Bb); -1(Dd)	NO
	T0-39	N.D.	NO		T0-34	N.D.	NO
	T0-47	N.D.	NO		T0-48	N.D.	YES
	T0-65	N.D.	YES		T0-53	N.D.	YES
	T0-73	N.D.	NO		T0-67	N.D.	NO
	T0-75	N.D.	NO		T0-72	N.D.	YES

N.A., not available, these mutant types were not obtained from the experiments. N.D., not detected.
[a]"–" indicates deletion of the indicated number of nucleotides; "+" indicates insertion of the indicated number of nucleotides; "–/+" indicates simultaneous deletion and insertion of the indicated number of nucleotides at the same site. [b] Based on the absence of mutant plants harboring CRISPR DNA construct or not.

Supplementary Table 4. Potential off-target sites for TaGASR7-sgRNA and TaGW2-sgRNA in wheat.

Target	Site name	Sequence	Forward primer sequence	Reverse primer sequence
Target 1 (*GASR7*)	GASR7	CCGCCGGGCACCTACGGCAAC		
	OT1-1	GTcGtCGTAGGTGCCCGGCGG	CTCGCTCAGGTTCCTGGGCTTCTT	CGGGTCCCATTATCCTCTCTCCTA
	OT1-2	GTTGCCGccGGTGCCCGGTGG	GTTCGTTCTCACGACGGTGGAGAC	TAGGTGCCCGTCTCTTTCGTACAG
	OT1-3	GgaGCCGTcGGTGCCCGGAGG	TTCCGATGGCCGAAACTCGACTTC	TTGTCGCTGCCTTCGCATCTCAAG
	OT1-4	GTcGCCGTccGTGCCCGGCGG	GAGGAGCTGATCCTCTGTTCAATC	GAAGGAGAGGATCCCCGAGCTGCT
	OT1-5	GTTcCCGTgGGcGCCCGGGGG	TCGCGTGTTGGTGCTTGGGAAG	TCCATGCTAACTTACACCCAATTG
	OT1-6	cTTGgCGTAcGTGCCCGGCGG	AAGTCCGCCGCATGCATGAGTTCC	GCACCTTGCAGTGACATATACTCC
	OT1-7	GTTcCCaTgGGTGCCCGGGGG	TGAGGTCTGGTCGCTACACGAA	GTGTGCCACTGAGATGTAGCAT
	OT1-8	GTTGaCcTtGGTGCCCGGCGG	ACTTGTCCTCGCCCTTCTGCTT	ATGGTCACTATCACCGACAAGA
	OT1-9	GTTGCaGcAcGTGCCCGGCGG	GGTTATCAAAGGTCTCCAACAACA	ATCGCTTACCGCTGTCAGAACG
	OT1-10	cTcGCCGTAtGTGCCCGGCGG	CGCTTCATCTTCAAGTCAATGTTC	TGGACTGGTGTTTACCCTTTGTT
	OT1-11	GTgGtCGgAGGTGCCCGGAGG	GGAACGATGGCAGTGACGATGA	TCCCTTTGTTCCCGCACTTAGT
	OT1-12	tTTGCCcTAGGaGCCCGGAGG	CATAAATGGAGTGAGGATGAACTG	CGTTTATTCTTACCAGGCGGAC
	OT1-13	GTTGaCGgAGaTGCCCGGAGG	ACGCTTCCTCTGGGCTCTTAGG	CAAACTTGCTGACCCACCTGTC
	OT1-14	cTTGtCGgAGaTGCCCGGCGG	CCTTCACTATCCGCCGTCCAGA	CGCATCAAACGCAGCAACTTGT
	OT1-15	GgcGCgcTAGGTGCCCGGCGG	GATGCCCAGAACAAGGAGGTCG	GCCGCACAGGTCGTAGTTGTCA
	OT1-16	GTTcaCcTtGGTGCCCGGCGG	GGTGTGGCATCATCATCATCATCT	ATCGTCACCATCACCGACAAGA
	OT1-17	GTcGCgcTAcGTGCCCGGCGG	AATGGACGCCCAGAACAAGGAG	AGGACGTAGGAGCTGAACGGCA
	OT1-18	GacGCgGTAGGaGCCCGaGGG	GAGGAGGAGGAACACGTTGTCC	AAAGCAAATGATGCCTCCCAGC
	OT1-19	GTcGaaGTgGGTGCgCGGCGG	CCATTGCCCTTCACCCACATCT	CCTTGTTATGCTGATGTATCGTGC
	OT1-20	cgTcgCGTAcGTGCCCGGCGG	CACCGTGTTCAAGGTCAAGGAC	GAAGTAAGTGGCGAGGCACCCT
	OT1-21	GgcGCaGgAGGaGCCCGGCGG	GGAAGGACGAGGAGAAGAAGAG	GAACTCGGACAGCATCTACGAC
	OT1-22	agaGaCGgAGGTGCCCGGCGG	AGGTCCTCACCGCAACTCGTAT	CGTTTGTGGACTGGCAGGAAAT
	OT1-23	GcTcCtGTAGcTGCgCGGCGG	CAGCGGCAGCCTCCATAAAGAG	CGCCGTTCAGTTGGCATACAAG
	OT1-24	GaTGtaGTAcGTcCCCGGCGG	GGTCTTTCACCCACCCGAACTC	CAACTACTGCCGATTTCGTCGT
Target 2 (*GW2*)	GW2	CCTCTAGAAATACCCCATCCTG		
	OT2-1	tAaGATGGGtTATTTCTAGAGG	AAACTCAGCGTAAATCTGGCG	CCGAGACAAACAATGGACCAA
	OT2-2	gAGGATGGGaTtTTTCTAGAGG	GATGACAACGGGTGGCAAGAG	TGCCATCACCTCCCTACATCG
	OT2-3	aAGaATGGGccATTTCTAGAGG	CCCTCTGGTAACTCTTCGTTC	TCTCTGATGTGCTATGTTCCA
	OT2-4	aAGaATGGGtcATTTCTAGAGG	CTGCCTCTTGTTACCATCCAC	TCATCATTTGCTTCCTCTCTG
	OT2-5	CgGcATGGaGTATaTCTAGAGG	AATACCAACTAAGCCTGAGCAAA	ACTCCCTAGACCACGAACGAC
	OT2-6	CcGGAgGGaGTATgTCTAGAGG	GCGTATTACATTCGTGTCAAGT	TCAAACAACAGTTACCGCAAC
	OT2-7	ttGGAaGGGGgATTTCTAGAGG	AAAGCAATGGGCACAAAGTCT	GTATTCGGTGATTTCTGCGTT
	OT2-8	gAGGAcaGaGgATTTCTAGAGG	GCAGAAGCAGCAACTAATAAGAAG	TCATTCAGAGCAGTCAGAAGC

Supplementary Table 5. Molecular and genetic analysis of CRISPR/Cas9-induced mutations in *TaGASR7*, *TaDEP1* and *TaLOX2* homoeologs and their transmission to the T1 generation.

Gene name	Wheat variety	Plant ID	Analysis of T0 plants			Segregation of mutations in the T1 generation					
			**Genotype of homoeologs**	**Mutation detected (bp)[a]**	**CRISPR-free**	**No. of tested plants**	**Wild type**	**Hetero**	**Homo**	**Mutation Transmission (%)[b]**	**CRISPR-free (%)[c]**
sg-GASR7	Bobwhite	T0-1	Aa	+1	YES	26	7 (AA)	13 (Aa)	6 (aa)	73.1[d]	100
			BB				26 (BB)	0 (Bb)	0 (bb)		
			dd	-1/+2			0 (DD)	0 (Dd)	26 (dd)	100	
		T0-5	aa	+1, -7/+15	YES	44	0 (AA)	0 (Aa)	44 (aa)	100	100
			bb	-1, -10			0 (BB)	0 (Bb)	44 (bb)	100	
			dd	+1, -37			0 (DD)	0 (Dd)	44 (dd)	100	
		T0-10	Aa	-1	YES	35	10 (AA)	16 (Aa)	9 (aa)	71.4[d]	100
			bb	+1			0 (BB)	0 (Bb)	35 (bb)	100	
			DD				35 (DD)	0 (Dd)	0 (dd)		
		T0-16	Aa	-6/+1	YES	30	9 (AA)	14 (Aa)	7 (aa)	70.0[d]	100
			bb	-25			0 (BB)	0 (Bb)	30 (bb)	100	
			dd	+1, -26			0 (DD)	0 (Dd)	30 (dd)	100	
		T0-20	Aa	-6	YES	29	8 (AA)	14 (Aa)	7(aa)	72.4[d]	100
			Bb	-17			7 (BB)	15 (Bb)	7(bb)	75.9[d]	
			Dd	+5			8 (DD)	15 (Dd)	6(dd)	72.4[d]	
		T0-36	Aa	+12	YES	29	9 (AA)	14 (Aa)	6 (aa)	69.0[d]	100
			BB				29 (BB)	0 (Bb)	0 (bb)		
			DD				29 (DD)	0 (Dd)	0 (dd)		
sg-LOX2	Kenong 199	T0-3	Dd	-1	YES	23	5 (DD)	14 (Dd)	4 (dd)	78.3[d]	100
		T0-16	Dd	-7	YES	42	8 (DD)	24 (Dd)	10 (dd)	81.0[d]	100
		T0-22	dd	-3, -5	YES	30	0 (DD)	0 (Dd)	30 (dd)	100	100
		T0-35	dd	-2, -9	YES	52	0 (DD)	0 (Dd)	52 (dd)	100	100
		T0-65	dd	+7,-10	YES	18	0 (DD)	0 (Dd)	18 (dd)	100	100
		T0-68	Dd	-11	YES	28	7 (DD)	16 (Dd)	5 (dd)	75.0[d]	100

Supplementary Table 5. Molecular and genetic analysis of CRISPR/Cas9-induced mutations in *TaGASR7*, *TaDEP1* and *TaLOX2* homoeologs and their transmission to the T1 generation (continued).

Gene name	Wheat variety	Plant ID	Analysis of T0 plants			Segregation of mutations in the T1 generation					
			Genotype of homoeologs	Mutation detected (bp)[a]	CRISPR-free	No. of tested plants	Wild type	Hetero	Homo	Mutation Transmission (%)[b]	CRISPR-free (%)[c]
sg-DEP1	Kenong 199	T0-2	Aa	-2	YES	3	1 (AA)	2 (Aa)	0 (aa)	66.7	100
			bb	+1			0 (BB)	0 (Bb)	3 (bb)	100	
			dd	+1, -1			0 (DD)	0 (Dd)	3 (dd)	100	
		T0-7	aa	-1, +1	YES	32	0 (AA)	0 (Aa)	32 (aa)	100	100
			Bb	+1			9 (BB)	16 (Bb)	7 (bb)	71.8[d]	
			dd	-1			0 (DD)	0 (Dd)	32 (dd)	100	
		T0-8	AA		YES	37	37 (AA)	0 (Aa)	0 (aa)		100
			Bb	-1			7 (BB)	24 (Bb)	6 (bb)	81.1[d]	
			DD				37 (DD)	0 (Dd)	0 (dd)		
		T0-12	Aa	-3	YES	18	4 (AA)	8 (Aa)	6 (aa)	77.8[d]	100
			Bb	+5			5 (BB)	10 (Bb)	3 (bb)	72.2[d]	
			DD				18 (DD)	0 (Dd)	0 (dd)		
		T0-14	AA		YES	17	17 (AA)	0 (Aa)	0 (aa)		100
			Bb	+2			4 (BB)	8 (Bb)	5 (bb)	76.5[d]	
			Dd	-1/+10			3 (DD)	10 (Dd)	4 (dd)	82.3[d]	
		T0-23	Aa	+1	YES	24	6 (AA)	14 (Aa)	4 (aa)	75.0[d]	100
			Bb	+4			6 (BB)	13 (Bb)	5 (bb)	75.0[d]	
			dd	-12, -16			0 (DD)	0 (Dd)	24 (dd)	100	

Hetero, heterozygous; Homo, homozygous.

[a] "–" indicates deletion of the indicated number of nucleotides; "+" indicates insertion of the indicated number of nucleotides; "–/+" indicates simultaneous deletion and insertion of the indicated number of nucleotides at the same site. [b] Based on the number of plants carrying the observed mutations over the total number of plants tested. [c] Based on the number of mutant plants not harboring CRISPR DNA over the total number of mutant plants tested. [d] Segregation of the heterozygous lines conforms to a Mendelian 1:2:1 ratio according to the $\chi2$ test ($P > 0.05$).
